# Trends in Mental and Physical Health-Related Quality of Life in Low-Income Older Persons in the United States, 2003 to 2017

**DOI:** 10.1001/jamanetworkopen.2019.17868

**Published:** 2019-12-18

**Authors:** David H. Rehkopf, Frank F. Furstenberg, John W. Rowe

**Affiliations:** 1 Stanford University School of Medicine, Palo Alto, California; 2 University of Pennsylvania, Philadelphia; 3 Columbia University Mailman School of Public Health, New York, New York

## Abstract

This cross-sectional study of respondents to the Behavioral Risk Factor Surveillance System survey assesses trends between 2003 and 2017 in mental and physical health-related quality of life in low-income US individuals 60 years or older.

## Introduction

Several studies have documented increases in morbidity and mortality^1^ and decreases in mental health status^2^ in the United States for those aged 35 to 59 years, which may also have occurred in older adults.^3^ This cross-sectional study examines time trends in general health and in physical and mental health measured in US adults aged 60 years and older.

## Methods

The study population data were drawn from the Behavioral Risk Factor Surveillance System, a telephone survey administered by state health departments with assistance from the Centers for Disease Control and Prevention. Verbal consent was obtained from participants contacted through randomly chosen telephone numbers. Personally identifiable information was not obtained and respondents were able to end the interview at any time. General, physical, and mental health were measures addressed in the survey.^4^ All individuals 60 years and older surveyed cross-sectionally between 2003 and 2017 who reported all 3 health measures (<3% missing) were included for a total sample size of 2 432 609. These cross-sectional samples were of the US population over time, not individuals followed over time. General health was assessed with the question, “Would you say that in general your health is” with response options of “excellent,” “very good,” “good,” “fair,” “poor,” “don’t know/not sure,” or “refused.” Physical health was assessed with the question, “Now thinking about your physical health, which includes physical illness and injury, for how many days during the past 30 days was your physical health not good?” with response options in number of days. Mental health was assessed with the question: “Now thinking about your mental health, which includes stress, depression, and problems with emotions, for how many days during the past 30 days was your mental health not good?” with response options in number of days.^5^ We estimated trends between cross-sectional measures of the 3 outcomes using generalized additive models with 3 *df*.^6^ Analyses were performed with R statistical software version 3.5.3 (R Foundation), and 2-tailed *P* < .05 was considered statistically significant. With the large sample size, 95% CIs and SEs were very small and therefore not reported. This study followed the Strengthening the Reporting of Observational Studies in Epidemiology (STROBE) reporting guideline for cross-sectional studies and, although considered nonhuman participant research, was approved by the Panel on Medical Human Subjects at Stanford University.

## Results

**Figure 1.**
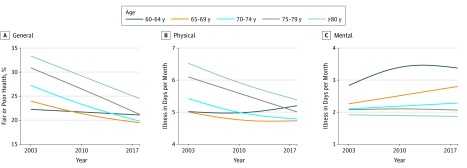
General, Physical, and Mental Health by Age Category, 2003 to 2017 The study population data were drawn from the Behavioral Risk Factor Surveillance System, a telephone survey administered by state health departments with assistance from the Centers for Disease Control and Prevention. Fair or poor health was assessed with the question “Would you say that in general your health is” with response options of “excellent,” “very good,” “good,” “fair,” “poor,” “don’t know/not sure,” or “refused.” Trends were modeled with yearly data using a generalized additive model with penalized splines with 3 *df*. The 95% CIs are extremely narrow because of the large sample size and thus are not depicted here.

In the study sample of 2 432 609 respondents, 62% were women, 6.5% were black individuals, 3.2% were Latinx individuals, 41% had a household income of less than or equal to $35 000 per year, and 10% of the sample had less than a high school education. These proportions were generally stable from 2003 to 2017. General health and physical health improved or were stable in all age groups 65 years and older from 2003 to 2017 (Figure 1). For example, the percentage of the population aged 65 to 69 years stating that they were in fair or poor general health was 23% for 2003 and 19% for 2017, and physical health, measured as days per month with illness, in those aged 65 to 69 years for 2003 was 4.9 days and for 2017 was 4.9 days. For individuals aged 60 to 64 years, trends were stable for general health (23% for 2003 and 23% for 2017) and slightly declined for physical health (5.0 days for 2003 and 5.4 days for 2017). A more substantial decrease in mental health problems, measured as days per month with illness, occurred for those in groupings aged 60 to 64 (2.9 days for 2003 and 3.6 days for 2017), 65 to 69 (2.3 days for 2003 and 3.0 days for 2017), and 70 to 74 (2.2 days for 2003 and 2.4 days for 2017). The groups aged 60 to 64 and 65 to 69 years had a significant decrease in mental health over this period (3.5% increase in number of mentally unhealthy days per year; *t* = 14.01 [*P* < .001]). The surveys did not include specific questions about memory decline, which may also be important.

**Figure 2.**
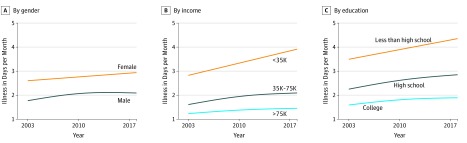
Mental Health Trends Among Individuals 60 Years and Older by Gender, Income, and Education, 2003 to 2017, Behavioral Risk Factor Surveillance System, 2003 to 2017 Trends were modeled with yearly data using a generalized additive model with penalized splines with 3 *df*. The 95% CIs are extremely narrow because of the large sample size and thus are not depicted here. Household income was assessed through self-report.

Mental health trends varied by gender, income, and education across the range of the population aged 60 years and older (Figure 2). Decreases in reported mental health, measured as days per month of illness, were similar for men and women, but greater for those with lower levels of income and those with less than a high school level of education. For example, number of days of poor mental health per month for those with household income below $35 000 per year were 2.9 days in 2003 to 4.1 days in 2017. For those with head of household not having a high school diploma, number of days of poor mental health per month were 3.6 days in 2003 to 4.4 days in 2017.

## Discussion

We found that individuals in groups aged 60 to 64 and 65 to 69 years shared a pattern of decreasing mental health well recognized in individuals aged 35 to 59, which may be owing in part to decreases in economic opportunity.^1^ We also found trends in decreases in mental health were greater for those with lower income or lower education than those with higher income or higher educational level. These decreases, however, began before the Great Recession of 2007 to 2009. Thus, while the decreases do not seem to be associated with the Great Recession, the mental health decreases (but not physical health trends) are shared with younger cohorts, even though 60- to 69-year-olds are primarily out of the labor force. The decreases may be because the effects of labor market conditions continue as cohorts age, especially for populations with low income and low levels of education, who exhibit both the highest burden as well as the greatest deterioration of mental health over time. Alternatively, they may be due to non–labor force–related issues. These trends will likely have important implications for future life expectancy, disability, and the capacity of older persons to engage productively in society.^7^
