# Wording Error in Results

**DOI:** 10.1001/jamanetworkopen.2019.21166

**Published:** 2020-01-22

In the Research Letter titled “Trends in Mental and Physical Health-Related Quality of Life in Low-Income Older Persons in the United States, 2003 to 2017,”^1^ published December 18, 2019, there was a wording error in the Results section. The sixth sentence should have read, “A more substantial decrease in mental health, measured as days per month with illness, occurred for those in groupings aged 60 to 64 (2.9 days for 2003 and 3.6 days for 2017), 65 to 69 (2.3 days for 2003 and 3.0 days for 2017), and 70 to 74 (2.2 days for 2003 and 2.4 days for 2017).” This article has been corrected.^1^
